# Benzyl­triphenyl­phospho­nium perchlorate

**DOI:** 10.1107/S1600536811021660

**Published:** 2011-06-11

**Authors:** Liwei Li, Xiaoqiang He

**Affiliations:** a College of Chemical Engineering and Pharmacy, Jingchu University of Technology, Jingmen, Hubei 448000, People’s Republic of China

## Abstract

The asymmetric unit of the title compound, C_25_H_22_P^+^·ClO_4_^−^, contains two independent cations and two independent anions. The closest inter­molecular contact is a weak inter­molecular C—H⋯π(arene) inter­action.

## Related literature

For the applications of large cations and anions, see: Fox *et al.* (2004 ▶); Huynh *et al.* (2000 ▶). For related structures, see: Zhang *et al.* (2010 ▶); Fischer & Wiebelhaus (1997 ▶); Hubner *et al.* (1997 ▶); Skapski & Stephens (1974 ▶).
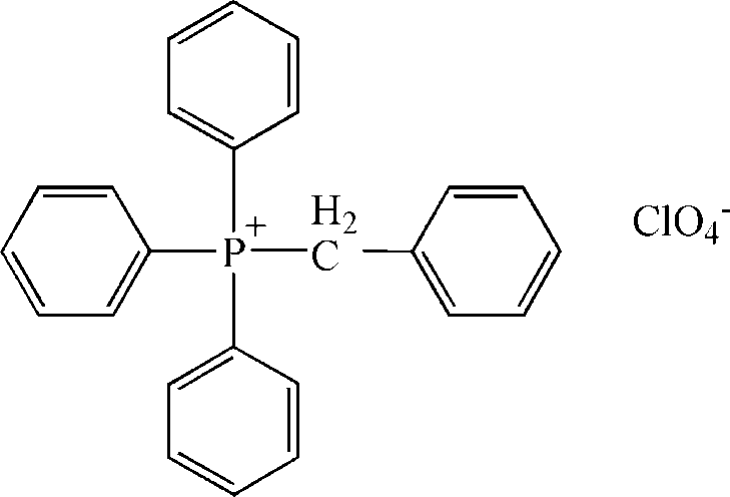


## Experimental

### 

#### Crystal data

C_25_H_22_P^+^·ClO_4_^−^
*M* *_r_* = 452.85
Triclinic, 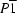

*a* = 10.096 (1) Å
*b* = 13.8967 (13) Å
*c* = 18.2577 (17) Å
α = 69.765 (2)°
β = 84.826 (2)°
γ = 73.195 (2)°
*V* = 2300.7 (4) Å^3^
*Z* = 4
Mo *K*α radiation
μ = 0.26 mm^−1^
*T* = 293 K
0.31 × 0.29 × 0.24 mm

#### Data collection

Bruker APEXII diffractometer
Absorption correction: multi-scan (*SADABS*; Sheldrick, 1996 ▶) *T*_min_ = 0.923, *T*_max_ = 0.939
11362 measured reflections
7992 independent reflections
5675 reflections with *I* > 2σ(*I*)
*R*_int_ = 0.019

#### Refinement

*R*[*F* ^2^ > 2σ(*F* ^2^)] = 0.060
*wR*(*F* ^2^) = 0.185
*S* = 1.02
7992 reflections
559 parameters
H-atom parameters constrained
Δρ_max_ = 0.95 e Å^−3^
Δρ_min_ = −0.36 e Å^−3^

### 

Data collection: *APEX2* (Bruker, 2004 ▶); cell refinement: *SAINT-Plus* (Bruker, 2001 ▶); data reduction: *SAINT-Plus*; program(s) used to solve structure: *SHELXS97* (Sheldrick, 2008 ▶); program(s) used to refine structure: *SHELXL97* (Sheldrick, 2008 ▶); molecular graphics: *SHELXTL* (Sheldrick, 2008 ▶); software used to prepare material for publication: *SHELXTL*.

## Supplementary Material

Crystal structure: contains datablock(s) I, global. DOI: 10.1107/S1600536811021660/lh5256sup1.cif

Structure factors: contains datablock(s) I. DOI: 10.1107/S1600536811021660/lh5256Isup2.hkl

Additional supplementary materials:  crystallographic information; 3D view; checkCIF report

## Figures and Tables

**Table 1:** Hydrogen-bond geometry (Å, °) *Cg* is the centroid of the C27–C32 ring.

*D*—H⋯*A*	*D*—H	H⋯*A*	*D*⋯*A*	*D*—H⋯*A*
C5—H5⋯*Cg*^i^	0.93	2.83	3.757 (9)	176

Symmetry code: (i) 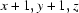.

**Fig. 1.**
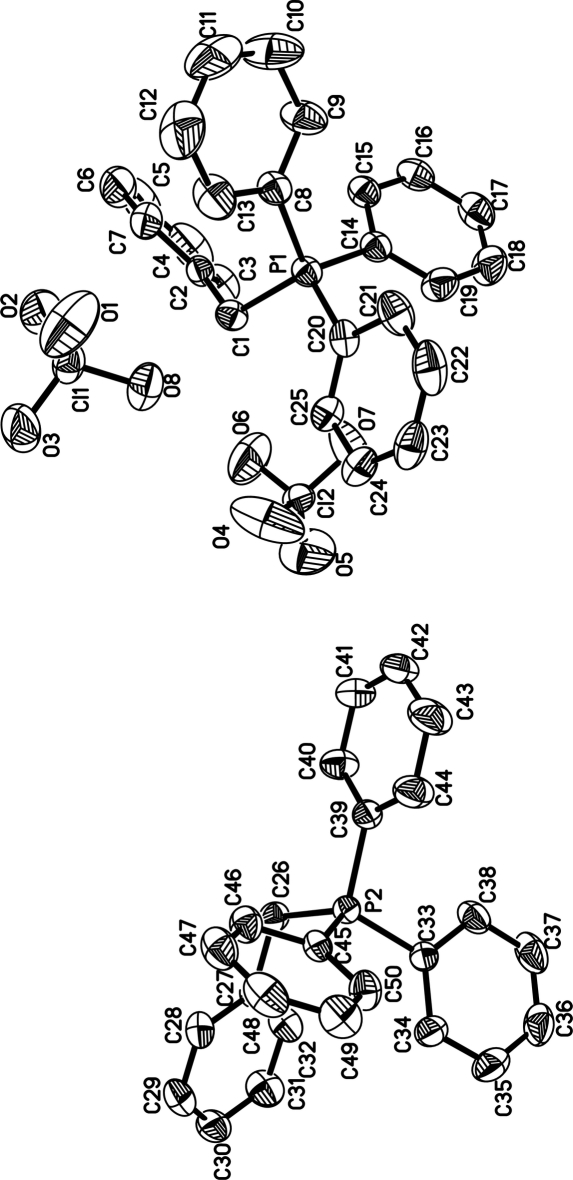
The asymmetric unit of the title compound. Displacement ellipsoids are drawn at the 30% probability level.

## supplementary crystallographic information

### Comment

Large cations and anions are often employed to act as counter ions in coordination chemistry (Fox *et al.*, 2004; Huynh *et al.*, 2000). Here, we report the crystal structure of the title compound.

The asymmetric unit of the title compound is shown in Fig. 1.  The P atoms are bonded in slightly distorted tetrahedral environments. The P—C bond distances are comparable to those in related componds containing Ph_3_(PhCH_2_)P^+^ cations (Zhang, *et al.*, 2010; Fischer & Wiebelhaus, 1997; Hubner, *et al.*, 1997; Skapski & Stephens, 1974). The closest intermolecular contact is a weak intermolecular C—H···π(arene) interaction. .

### Experimental

The title compound was synthesized by reacting [Ph_3_(PhCH_2_)]Cl and NaClO_4_.H_2_O(1:1, molar ratio) in ethanol. The mixture was stirred for for about 10 min at room temperature, then filtered, and then the filtrate was allowed to slowly evaporate undisturbed for ten days to afford colorless crystals suitable for X-ray diffraction with a yield about 85%.

### Refinement

H atoms were placed using the HFIX commands in *SHELXL-97* (Sheldrick, 2008) with C—H distances of 0.93 and 0.97 Å and were allowed for as riding atoms with *U*_iso_(H) = 1.2*U*_eq_(C).

### Crystal data

**Table:**

C_25_H_22_P^+^·ClO_4_^−^	*Z* = 4
*M**_r_* = 452.85	*F*(000) = 944
Triclinic, *P*1	*D*_x_ = 1.307 Mg m^−^^3^
Hall symbol: -P 1	Mo *K*α radiation, λ = 0.71073 Å
*a* = 10.096 (1) Å	Cell parameters from 2162 reflections
*b* = 13.8967 (13) Å	θ = 2.4–26.7°
*c* = 18.2577 (17) Å	µ = 0.26 mm^−^^1^
α = 69.765 (2)°	*T* = 293 K
β = 84.826 (2)°	Block, colourless
γ = 73.195 (2)°	0.31 × 0.29 × 0.24 mm
*V* = 2300.7 (4) Å^3^	

### Data collection

**Table:**

Bruker APEXII diffractometer	7992 independent reflections
Radiation source: fine-focus sealed tube	5675 reflections with *I* > 2σ(*I*)
graphite	*R*_int_ = 0.019
φ and ω scans	θ_max_ = 25.0°, θ_min_ = 2.1°
Absorption correction: multi-scan (*SADABS*; Sheldrick, 1996)	*h* = −12→10
*T*_min_ = 0.923, *T*_max_ = 0.939	*k* = −16→16
11362 measured reflections	*l* = −21→21

### Refinement

**Table:**

Refinement on *F*^2^	Primary atom site location: structure-invariant direct methods
Least-squares matrix: full	Secondary atom site location: difference Fourier map
*R*[*F*^2^ > 2σ(*F*^2^)] = 0.060	Hydrogen site location: inferred from neighbouring sites
*wR*(*F*^2^) = 0.185	H-atom parameters constrained
*S* = 1.02	*w* = 1/[σ^2^(*F*_o_^2^) + (0.099*P*)^2^ + 1.1578*P*] where *P* = (*F*_o_^2^ + 2*F*_c_^2^)/3
7992 reflections	(Δ/σ)_max_ < 0.001
559 parameters	Δρ_max_ = 0.95 e Å^−^^3^
0 restraints	Δρ_min_ = −0.36 e Å^−^^3^

### Special details

**Table:**

Geometry. All e.s.d.'s (except the e.s.d. in the dihedral angle between two l.s. planes) are estimated using the full covariance matrix. The cell e.s.d.'s are taken into account individually in the estimation of e.s.d.'s in distances, angles and torsion angles; correlations between e.s.d.'s in cell parameters are only used when they are defined by crystal symmetry. An approximate (isotropic) treatment of cell e.s.d.'s is used for estimating e.s.d.'s involving l.s. planes.
Refinement. Refinement of *F*^2^ against ALL reflections. The weighted *R*-factor *wR* and goodness of fit *S* are based on *F*^2^, conventional *R*-factors *R* are based on *F*, with *F* set to zero for negative *F*^2^. The threshold expression of *F*^2^ > σ(*F*^2^) is used only for calculating *R*-factors(gt) *etc*. and is not relevant to the choice of reflections for refinement. *R*-factors based on *F*^2^ are statistically about twice as large as those based on *F*, and *R*- factors based on ALL data will be even larger.

### Fractional atomic coordinates and isotropic or equivalent isotropic displacement parameters (Å^2^)

**Table:**

	*x*	*y*	*z*	*U*_iso_*/*U*_eq_	
Cl1	0.28935 (10)	0.87956 (7)	0.37917 (6)	0.0715 (3)	
Cl2	0.64016 (9)	0.63901 (7)	0.12239 (5)	0.0618 (3)	
P1	0.78887 (9)	0.70653 (7)	0.35120 (5)	0.0504 (2)	
P2	0.15337 (8)	0.25542 (6)	0.15262 (5)	0.0455 (2)	
O1	0.2871 (6)	0.8624 (3)	0.4591 (2)	0.160 (2)	
O2	0.3355 (3)	0.9704 (2)	0.3405 (2)	0.1007 (10)	
O3	0.1552 (4)	0.8940 (3)	0.3548 (3)	0.1497 (19)	
O4	0.5282 (5)	0.6397 (6)	0.1692 (2)	0.186 (3)	
O5	0.6241 (5)	0.6110 (5)	0.0585 (3)	0.1600 (19)	
O6	0.6364 (6)	0.7454 (3)	0.0897 (4)	0.190 (2)	
O7	0.7664 (4)	0.5984 (5)	0.1599 (3)	0.190 (3)	
O8	0.3807 (3)	0.7868 (2)	0.3676 (2)	0.0951 (9)	
C1	0.6743 (3)	0.7966 (3)	0.27025 (19)	0.0552 (8)	
H1A	0.6667	0.7578	0.2365	0.066*	
H1B	0.5830	0.8198	0.2906	0.066*	
C2	0.7194 (3)	0.8937 (3)	0.2221 (2)	0.0540 (8)	
C3	0.7893 (4)	0.8939 (4)	0.1541 (2)	0.0749 (11)	
H3	0.8134	0.8331	0.1398	0.090*	
C4	0.8242 (6)	0.9860 (6)	0.1063 (3)	0.122 (2)	
H4	0.8721	0.9865	0.0603	0.147*	
C5	0.7882 (7)	1.0740 (6)	0.1274 (6)	0.152 (4)	
H5	0.8101	1.1354	0.0950	0.183*	
C6	0.7216 (7)	1.0744 (4)	0.1938 (5)	0.129 (2)	
H6	0.6995	1.1357	0.2075	0.155*	
C7	0.6850 (5)	0.9851 (3)	0.2426 (3)	0.0867 (13)	
H7	0.6379	0.9863	0.2886	0.104*	
C8	0.7901 (4)	0.7712 (3)	0.42106 (19)	0.0606 (9)	
C9	0.9078 (5)	0.7808 (4)	0.4455 (2)	0.0905 (14)	
H9	0.9937	0.7507	0.4277	0.109*	
C10	0.8984 (8)	0.8365 (6)	0.4974 (3)	0.130 (2)	
H10	0.9781	0.8449	0.5133	0.156*	
C11	0.7763 (10)	0.8775 (6)	0.5244 (3)	0.133 (2)	
H11	0.7719	0.9134	0.5595	0.160*	
C12	0.6574 (8)	0.8679 (6)	0.5014 (3)	0.138 (3)	
H12	0.5726	0.8974	0.5205	0.166*	
C13	0.6636 (5)	0.8135 (5)	0.4492 (3)	0.1033 (17)	
H13	0.5832	0.8060	0.4336	0.124*	
C14	0.9621 (3)	0.6633 (3)	0.31777 (19)	0.0557 (8)	
C15	1.0440 (4)	0.7335 (3)	0.2902 (2)	0.0623 (9)	
H15	1.0087	0.8041	0.2880	0.075*	
C16	1.1773 (4)	0.6984 (4)	0.2661 (2)	0.0709 (10)	
H16	1.2330	0.7447	0.2495	0.085*	
C17	1.2275 (4)	0.5957 (4)	0.2666 (2)	0.0825 (12)	
H17	1.3168	0.5727	0.2494	0.099*	
C18	1.1472 (5)	0.5264 (4)	0.2923 (3)	0.0894 (13)	
H18	1.1823	0.4566	0.2926	0.107*	
C19	1.0149 (4)	0.5594 (3)	0.3177 (3)	0.0760 (11)	
H19	0.9608	0.5120	0.3349	0.091*	
C20	0.7226 (3)	0.5925 (3)	0.39688 (19)	0.0554 (8)	
C21	0.7725 (4)	0.5226 (4)	0.4711 (2)	0.0810 (12)	
H21	0.8367	0.5360	0.4970	0.097*	
C22	0.7246 (5)	0.4330 (4)	0.5054 (3)	0.0917 (15)	
H22	0.7571	0.3862	0.5547	0.110*	
C23	0.6308 (5)	0.4131 (3)	0.4677 (3)	0.0820 (13)	
H23	0.6006	0.3523	0.4908	0.098*	
C24	0.5816 (4)	0.4814 (3)	0.3967 (2)	0.0714 (10)	
H24	0.5161	0.4679	0.3717	0.086*	
C25	0.6269 (4)	0.5711 (3)	0.3606 (2)	0.0590 (8)	
H25	0.5925	0.6171	0.3115	0.071*	
C26	0.0749 (4)	0.3622 (3)	0.06520 (19)	0.0562 (8)	
H26A	0.0360	0.4268	0.0781	0.067*	
H26B	0.1468	0.3749	0.0271	0.067*	
C27	−0.0372 (3)	0.3416 (2)	0.02821 (18)	0.0512 (8)	
C28	−0.1743 (4)	0.3715 (3)	0.0479 (2)	0.0628 (9)	
H28	−0.1990	0.4019	0.0870	0.075*	
C29	−0.2753 (4)	0.3568 (3)	0.0101 (2)	0.0731 (11)	
H29	−0.3677	0.3783	0.0233	0.088*	
C30	−0.2404 (4)	0.3103 (3)	−0.0472 (2)	0.0757 (11)	
H30	−0.3084	0.2999	−0.0724	0.091*	
C31	−0.1051 (5)	0.2801 (4)	−0.0662 (2)	0.0838 (12)	
H31	−0.0809	0.2496	−0.1052	0.101*	
C32	−0.0035 (4)	0.2938 (3)	−0.0288 (2)	0.0698 (10)	
H32	0.0886	0.2709	−0.0417	0.084*	
C33	0.2236 (3)	0.1339 (2)	0.13166 (18)	0.0489 (7)	
C34	0.1405 (4)	0.0721 (3)	0.1291 (2)	0.0665 (9)	
H34	0.0462	0.0928	0.1387	0.080*	
C35	0.1983 (5)	−0.0215 (3)	0.1121 (3)	0.0847 (12)	
H35	0.1432	−0.0647	0.1120	0.102*	
C36	0.3346 (6)	−0.0497 (4)	0.0957 (3)	0.0930 (15)	
H36	0.3720	−0.1120	0.0838	0.112*	
C37	0.4177 (5)	0.0113 (4)	0.0964 (3)	0.0917 (15)	
H37	0.5112	−0.0094	0.0850	0.110*	
C38	0.3631 (4)	0.1051 (3)	0.1140 (2)	0.0705 (10)	
H38	0.4193	0.1477	0.1140	0.085*	
C39	0.2891 (3)	0.2915 (3)	0.18479 (19)	0.0527 (8)	
C40	0.3374 (4)	0.3763 (3)	0.1383 (2)	0.0707 (10)	
H40	0.3000	0.4159	0.0886	0.085*	
C41	0.4405 (4)	0.4020 (4)	0.1654 (3)	0.0766 (11)	
H41	0.4723	0.4593	0.1340	0.092*	
C42	0.4957 (4)	0.3453 (4)	0.2366 (3)	0.0829 (12)	
H42	0.5657	0.3633	0.2542	0.099*	
C43	0.4493 (5)	0.2603 (4)	0.2841 (3)	0.1044 (16)	
H43	0.4878	0.2213	0.3336	0.125*	
C44	0.3452 (5)	0.2332 (4)	0.2579 (2)	0.0835 (12)	
H44	0.3137	0.1759	0.2896	0.100*	
C45	0.0268 (3)	0.2423 (2)	0.22866 (18)	0.0488 (7)	
C46	−0.0568 (4)	0.3353 (3)	0.2398 (2)	0.0644 (9)	
H46	−0.0501	0.4017	0.2065	0.077*	
C47	−0.1488 (4)	0.3286 (4)	0.3002 (3)	0.0777 (12)	
H47	−0.2051	0.3905	0.3075	0.093*	
C48	−0.1582 (4)	0.2316 (4)	0.3495 (2)	0.0825 (13)	
H48	−0.2206	0.2277	0.3905	0.099*	
C49	−0.0760 (4)	0.1392 (4)	0.3391 (2)	0.0806 (12)	
H49	−0.0838	0.0733	0.3726	0.097*	
C50	0.0186 (4)	0.1442 (3)	0.2786 (2)	0.0649 (9)	
H50	0.0756	0.0820	0.2720	0.078*	

### Atomic displacement parameters (Å^2^)

**Table:**

	*U*^11^	*U*^22^	*U*^33^	*U*^12^	*U*^13^	*U*^23^
Cl1	0.0651 (6)	0.0486 (5)	0.0899 (7)	−0.0150 (4)	0.0148 (5)	−0.0140 (5)
Cl2	0.0601 (5)	0.0640 (5)	0.0710 (6)	−0.0234 (4)	0.0099 (4)	−0.0318 (4)
P1	0.0498 (5)	0.0578 (5)	0.0429 (4)	−0.0128 (4)	−0.0004 (3)	−0.0176 (4)
P2	0.0456 (4)	0.0445 (4)	0.0463 (4)	−0.0120 (3)	−0.0011 (3)	−0.0151 (3)
O1	0.264 (6)	0.110 (3)	0.104 (3)	−0.054 (3)	0.062 (3)	−0.046 (2)
O2	0.096 (2)	0.0658 (18)	0.127 (3)	−0.0368 (16)	0.0062 (19)	−0.0054 (17)
O3	0.071 (2)	0.083 (2)	0.282 (6)	−0.0226 (18)	−0.009 (3)	−0.042 (3)
O4	0.129 (3)	0.391 (8)	0.104 (3)	−0.143 (5)	0.050 (3)	−0.118 (4)
O5	0.181 (4)	0.223 (5)	0.132 (3)	−0.067 (4)	0.037 (3)	−0.127 (4)
O6	0.203 (5)	0.078 (3)	0.278 (7)	−0.031 (3)	−0.013 (5)	−0.047 (4)
O7	0.087 (3)	0.207 (5)	0.190 (5)	0.000 (3)	−0.034 (3)	0.015 (4)
O8	0.091 (2)	0.0691 (18)	0.115 (2)	−0.0128 (15)	0.0239 (18)	−0.0320 (17)
C1	0.057 (2)	0.059 (2)	0.0480 (18)	−0.0189 (16)	−0.0023 (15)	−0.0130 (15)
C2	0.0462 (18)	0.0493 (18)	0.0586 (19)	−0.0103 (14)	−0.0053 (15)	−0.0094 (15)
C3	0.060 (2)	0.094 (3)	0.053 (2)	−0.022 (2)	0.0016 (17)	−0.002 (2)
C4	0.079 (3)	0.145 (6)	0.090 (4)	−0.041 (4)	−0.006 (3)	0.035 (4)
C5	0.086 (4)	0.096 (5)	0.205 (9)	−0.046 (4)	−0.047 (5)	0.064 (5)
C6	0.102 (4)	0.056 (3)	0.213 (8)	−0.024 (3)	−0.040 (5)	−0.015 (4)
C7	0.076 (3)	0.063 (3)	0.117 (4)	−0.010 (2)	−0.011 (2)	−0.031 (3)
C8	0.063 (2)	0.076 (2)	0.0439 (17)	−0.0122 (18)	−0.0019 (15)	−0.0258 (17)
C9	0.082 (3)	0.140 (4)	0.073 (3)	−0.036 (3)	0.001 (2)	−0.059 (3)
C10	0.153 (6)	0.200 (7)	0.095 (4)	−0.084 (5)	0.016 (4)	−0.096 (5)
C11	0.210 (8)	0.136 (5)	0.085 (4)	−0.052 (5)	0.008 (5)	−0.073 (4)
C12	0.155 (6)	0.154 (6)	0.094 (4)	0.019 (5)	0.005 (4)	−0.079 (4)
C13	0.082 (3)	0.151 (5)	0.082 (3)	0.000 (3)	0.002 (2)	−0.071 (3)
C14	0.0532 (19)	0.067 (2)	0.0485 (18)	−0.0123 (16)	−0.0010 (14)	−0.0246 (16)
C15	0.060 (2)	0.072 (2)	0.055 (2)	−0.0162 (18)	−0.0025 (16)	−0.0214 (18)
C16	0.060 (2)	0.096 (3)	0.062 (2)	−0.030 (2)	0.0039 (18)	−0.025 (2)
C17	0.056 (2)	0.114 (4)	0.076 (3)	−0.011 (2)	0.008 (2)	−0.042 (3)
C18	0.075 (3)	0.086 (3)	0.114 (4)	−0.012 (2)	0.022 (3)	−0.055 (3)
C19	0.069 (2)	0.082 (3)	0.093 (3)	−0.027 (2)	0.019 (2)	−0.049 (2)
C20	0.0523 (19)	0.0539 (19)	0.0472 (17)	−0.0061 (15)	0.0060 (14)	−0.0095 (15)
C21	0.070 (3)	0.087 (3)	0.062 (2)	−0.011 (2)	−0.0063 (19)	−0.002 (2)
C22	0.083 (3)	0.074 (3)	0.069 (3)	−0.002 (2)	0.010 (2)	0.018 (2)
C23	0.083 (3)	0.052 (2)	0.091 (3)	−0.013 (2)	0.031 (3)	−0.011 (2)
C24	0.080 (3)	0.055 (2)	0.079 (3)	−0.0220 (19)	0.020 (2)	−0.025 (2)
C25	0.069 (2)	0.0526 (19)	0.0527 (19)	−0.0155 (17)	0.0056 (16)	−0.0166 (16)
C26	0.065 (2)	0.0484 (18)	0.0510 (18)	−0.0173 (16)	−0.0062 (15)	−0.0091 (15)
C27	0.0562 (19)	0.0437 (17)	0.0445 (17)	−0.0099 (14)	−0.0061 (14)	−0.0050 (13)
C28	0.062 (2)	0.059 (2)	0.057 (2)	−0.0007 (17)	−0.0044 (17)	−0.0189 (17)
C29	0.052 (2)	0.081 (3)	0.070 (2)	−0.0072 (19)	−0.0079 (18)	−0.014 (2)
C30	0.072 (3)	0.079 (3)	0.074 (3)	−0.020 (2)	−0.025 (2)	−0.017 (2)
C31	0.086 (3)	0.110 (3)	0.068 (3)	−0.021 (3)	−0.010 (2)	−0.047 (2)
C32	0.059 (2)	0.097 (3)	0.054 (2)	−0.013 (2)	0.0014 (17)	−0.034 (2)
C33	0.0496 (18)	0.0500 (17)	0.0473 (17)	−0.0107 (14)	−0.0012 (13)	−0.0186 (14)
C34	0.063 (2)	0.060 (2)	0.084 (3)	−0.0208 (18)	0.0059 (19)	−0.0319 (19)
C35	0.098 (3)	0.067 (3)	0.105 (3)	−0.028 (2)	0.002 (3)	−0.044 (2)
C36	0.099 (4)	0.072 (3)	0.105 (3)	0.015 (3)	−0.026 (3)	−0.052 (3)
C37	0.060 (2)	0.114 (4)	0.106 (3)	0.015 (2)	−0.013 (2)	−0.071 (3)
C38	0.0455 (19)	0.090 (3)	0.086 (3)	−0.0079 (18)	−0.0018 (18)	−0.049 (2)
C39	0.0507 (18)	0.0539 (19)	0.0576 (19)	−0.0124 (15)	−0.0020 (15)	−0.0248 (16)
C40	0.077 (3)	0.079 (3)	0.063 (2)	−0.040 (2)	−0.0002 (19)	−0.017 (2)
C41	0.076 (3)	0.085 (3)	0.085 (3)	−0.041 (2)	0.001 (2)	−0.033 (2)
C42	0.064 (2)	0.094 (3)	0.110 (4)	−0.026 (2)	−0.011 (2)	−0.051 (3)
C43	0.103 (4)	0.111 (4)	0.094 (3)	−0.038 (3)	−0.047 (3)	−0.009 (3)
C44	0.090 (3)	0.082 (3)	0.077 (3)	−0.040 (2)	−0.026 (2)	−0.004 (2)
C45	0.0440 (17)	0.0532 (18)	0.0451 (16)	−0.0082 (14)	−0.0045 (13)	−0.0147 (14)
C46	0.060 (2)	0.067 (2)	0.066 (2)	−0.0085 (17)	0.0042 (17)	−0.0300 (18)
C47	0.060 (2)	0.099 (3)	0.076 (3)	−0.005 (2)	0.007 (2)	−0.046 (3)
C48	0.061 (2)	0.128 (4)	0.059 (2)	−0.023 (3)	0.0150 (19)	−0.038 (3)
C49	0.080 (3)	0.093 (3)	0.058 (2)	−0.029 (2)	0.008 (2)	−0.009 (2)
C50	0.064 (2)	0.061 (2)	0.055 (2)	−0.0069 (17)	0.0026 (17)	−0.0105 (17)

### Geometric parameters (Å, °)

**Table:**

Cl1—O1	1.394 (4)	C21—H21	0.9300
Cl1—O3	1.401 (4)	C22—C23	1.361 (7)
Cl1—O2	1.405 (3)	C22—H22	0.9300
Cl1—O8	1.422 (3)	C23—C24	1.349 (6)
Cl2—O4	1.354 (4)	C23—H23	0.9300
Cl2—O7	1.373 (4)	C24—C25	1.380 (5)
Cl2—O6	1.380 (4)	C24—H24	0.9300
Cl2—O5	1.386 (4)	C25—H25	0.9300
P1—C8	1.799 (3)	C26—C27	1.510 (5)
P1—C14	1.802 (3)	C26—H26A	0.9700
P1—C20	1.803 (4)	C26—H26B	0.9700
P1—C1	1.811 (3)	C27—C28	1.378 (5)
P2—C33	1.791 (3)	C27—C32	1.386 (5)
P2—C39	1.794 (3)	C28—C29	1.380 (5)
P2—C45	1.798 (3)	C28—H28	0.9300
P2—C26	1.813 (3)	C29—C30	1.379 (6)
C1—C2	1.503 (5)	C29—H29	0.9300
C1—H1A	0.9700	C30—C31	1.359 (6)
C1—H1B	0.9700	C30—H30	0.9300
C2—C3	1.371 (5)	C31—C32	1.373 (5)
C2—C7	1.388 (5)	C31—H31	0.9300
C3—C4	1.399 (7)	C32—H32	0.9300
C3—H3	0.9300	C33—C34	1.377 (5)
C4—C5	1.348 (11)	C33—C38	1.389 (5)
C4—H4	0.9300	C34—C35	1.391 (5)
C5—C6	1.333 (11)	C34—H34	0.9300
C5—H5	0.9300	C35—C36	1.354 (6)
C6—C7	1.382 (8)	C35—H35	0.9300
C6—H6	0.9300	C36—C37	1.358 (7)
C7—H7	0.9300	C36—H36	0.9300
C8—C9	1.364 (5)	C37—C38	1.396 (5)
C8—C13	1.377 (5)	C37—H37	0.9300
C9—C10	1.399 (7)	C38—H38	0.9300
C9—H9	0.9300	C39—C44	1.371 (5)
C10—C11	1.327 (9)	C39—C40	1.383 (5)
C10—H10	0.9300	C40—C41	1.375 (5)
C11—C12	1.362 (9)	C40—H40	0.9300
C11—H11	0.9300	C41—C42	1.337 (6)
C12—C13	1.394 (7)	C41—H41	0.9300
C12—H12	0.9300	C42—C43	1.381 (6)
C13—H13	0.9300	C42—H42	0.9300
C14—C19	1.387 (5)	C43—C44	1.385 (6)
C14—C15	1.392 (5)	C43—H43	0.9300
C15—C16	1.381 (5)	C44—H44	0.9300
C15—H15	0.9300	C45—C50	1.374 (5)
C16—C17	1.366 (6)	C45—C46	1.395 (4)
C16—H16	0.9300	C46—C47	1.372 (5)
C17—C18	1.370 (6)	C46—H46	0.9300
C17—H17	0.9300	C47—C48	1.362 (6)
C18—C19	1.375 (5)	C47—H47	0.9300
C18—H18	0.9300	C48—C49	1.378 (6)
C19—H19	0.9300	C48—H48	0.9300
C20—C25	1.371 (5)	C49—C50	1.389 (5)
C20—C21	1.401 (5)	C49—H49	0.9300
C21—C22	1.390 (6)	C50—H50	0.9300
			
O1—Cl1—O3	108.3 (3)	C23—C22—C21	120.6 (4)
O1—Cl1—O2	108.6 (3)	C23—C22—H22	119.7
O3—Cl1—O2	111.0 (2)	C21—C22—H22	119.7
O1—Cl1—O8	107.3 (3)	C24—C23—C22	120.1 (4)
O3—Cl1—O8	110.1 (2)	C24—C23—H23	120.0
O2—Cl1—O8	111.4 (2)	C22—C23—H23	120.0
O4—Cl2—O7	115.8 (3)	C23—C24—C25	121.0 (4)
O4—Cl2—O6	103.7 (4)	C23—C24—H24	119.5
O7—Cl2—O6	100.5 (4)	C25—C24—H24	119.5
O4—Cl2—O5	112.0 (3)	C20—C25—C24	120.2 (3)
O7—Cl2—O5	118.6 (4)	C20—C25—H25	119.9
O6—Cl2—O5	103.3 (4)	C24—C25—H25	119.9
C8—P1—C14	109.83 (16)	C27—C26—P2	114.9 (2)
C8—P1—C20	109.12 (16)	C27—C26—H26A	108.5
C14—P1—C20	109.48 (16)	P2—C26—H26A	108.5
C8—P1—C1	109.95 (17)	C27—C26—H26B	108.5
C14—P1—C1	110.92 (15)	P2—C26—H26B	108.5
C20—P1—C1	107.50 (16)	H26A—C26—H26B	107.5
C33—P2—C39	109.73 (15)	C28—C27—C32	118.3 (3)
C33—P2—C45	111.81 (15)	C28—C27—C26	121.8 (3)
C39—P2—C45	107.62 (15)	C32—C27—C26	119.8 (3)
C33—P2—C26	109.90 (15)	C27—C28—C29	120.5 (3)
C39—P2—C26	108.29 (16)	C27—C28—H28	119.8
C45—P2—C26	109.41 (15)	C29—C28—H28	119.8
C2—C1—P1	114.7 (2)	C30—C29—C28	120.5 (4)
C2—C1—H1A	108.6	C30—C29—H29	119.8
P1—C1—H1A	108.6	C28—C29—H29	119.8
C2—C1—H1B	108.6	C31—C30—C29	119.0 (4)
P1—C1—H1B	108.6	C31—C30—H30	120.5
H1A—C1—H1B	107.6	C29—C30—H30	120.5
C3—C2—C7	119.2 (4)	C30—C31—C32	121.1 (4)
C3—C2—C1	119.2 (3)	C30—C31—H31	119.5
C7—C2—C1	121.5 (3)	C32—C31—H31	119.5
C2—C3—C4	119.8 (5)	C31—C32—C27	120.6 (4)
C2—C3—H3	120.1	C31—C32—H32	119.7
C4—C3—H3	120.1	C27—C32—H32	119.7
C5—C4—C3	119.6 (6)	C34—C33—C38	120.0 (3)
C5—C4—H4	120.2	C34—C33—P2	121.1 (3)
C3—C4—H4	120.2	C38—C33—P2	118.8 (3)
C6—C5—C4	121.1 (6)	C33—C34—C35	119.6 (4)
C6—C5—H5	119.4	C33—C34—H34	120.2
C4—C5—H5	119.4	C35—C34—H34	120.2
C5—C6—C7	121.0 (7)	C36—C35—C34	120.1 (4)
C5—C6—H6	119.5	C36—C35—H35	119.9
C7—C6—H6	119.5	C34—C35—H35	119.9
C6—C7—C2	119.2 (5)	C35—C36—C37	121.1 (4)
C6—C7—H7	120.4	C35—C36—H36	119.4
C2—C7—H7	120.4	C37—C36—H36	119.4
C9—C8—C13	120.0 (4)	C36—C37—C38	120.1 (4)
C9—C8—P1	123.3 (3)	C36—C37—H37	119.9
C13—C8—P1	116.7 (3)	C38—C37—H37	119.9
C8—C9—C10	119.4 (5)	C33—C38—C37	119.0 (4)
C8—C9—H9	120.3	C33—C38—H38	120.5
C10—C9—H9	120.3	C37—C38—H38	120.5
C11—C10—C9	120.5 (6)	C44—C39—C40	119.5 (3)
C11—C10—H10	119.8	C44—C39—P2	118.6 (3)
C9—C10—H10	119.8	C40—C39—P2	121.9 (3)
C10—C11—C12	121.2 (5)	C41—C40—C39	120.1 (4)
C10—C11—H11	119.4	C41—C40—H40	120.0
C12—C11—H11	119.4	C39—C40—H40	120.0
C11—C12—C13	119.6 (6)	C42—C41—C40	120.6 (4)
C11—C12—H12	120.2	C42—C41—H41	119.7
C13—C12—H12	120.2	C40—C41—H41	119.7
C8—C13—C12	119.4 (5)	C41—C42—C43	120.4 (4)
C8—C13—H13	120.3	C41—C42—H42	119.8
C12—C13—H13	120.3	C43—C42—H42	119.8
C19—C14—C15	118.9 (3)	C42—C43—C44	119.9 (4)
C19—C14—P1	120.1 (3)	C42—C43—H43	120.1
C15—C14—P1	121.0 (3)	C44—C43—H43	120.1
C16—C15—C14	120.1 (4)	C39—C44—C43	119.5 (4)
C16—C15—H15	119.9	C39—C44—H44	120.2
C14—C15—H15	119.9	C43—C44—H44	120.2
C17—C16—C15	120.0 (4)	C50—C45—C46	120.2 (3)
C17—C16—H16	120.0	C50—C45—P2	121.8 (2)
C15—C16—H16	120.0	C46—C45—P2	117.8 (3)
C16—C17—C18	120.4 (4)	C47—C46—C45	119.7 (4)
C16—C17—H17	119.8	C47—C46—H46	120.1
C18—C17—H17	119.8	C45—C46—H46	120.1
C17—C18—C19	120.4 (4)	C48—C47—C46	120.4 (4)
C17—C18—H18	119.8	C48—C47—H47	119.8
C19—C18—H18	119.8	C46—C47—H47	119.8
C18—C19—C14	120.1 (4)	C47—C48—C49	120.4 (4)
C18—C19—H19	120.0	C47—C48—H48	119.8
C14—C19—H19	120.0	C49—C48—H48	119.8
C25—C20—C21	119.0 (3)	C48—C49—C50	120.2 (4)
C25—C20—P1	122.0 (3)	C48—C49—H49	119.9
C21—C20—P1	118.9 (3)	C50—C49—H49	119.9
C22—C21—C20	119.0 (4)	C45—C50—C49	119.2 (4)
C22—C21—H21	120.5	C45—C50—H50	120.4
C20—C21—H21	120.5	C49—C50—H50	120.4

### Hydrogen-bond geometry (Å, °)

**Table:**

Cg is the centroid of the C27–C32 ring.

**Table:**

*D*—H···*A*	*D*—H	H···*A*	*D*···*A*	*D*—H···*A*
C5—H5···Cg^i^	0.93	2.83	3.757 (9)	176

Symmetry codes: (i) *x*+1, *y*+1, *z*.
